# Evaluation of the drug solubility and rush ageing on drug release performance of various model drugs from the modified release polyethylene oxide matrix tablets

**DOI:** 10.1007/s13346-016-0344-5

**Published:** 2016-11-21

**Authors:** Saeed Shojaee, Ali Nokhodchi, Mohammed Maniruzzaman

**Affiliations:** 1 Medway School of Pharmacy, University of Kent, Chatham Maritime, Chatham, ME4 4TB UK; 2 Hamadan University of Medical Science, Daneshgah-e-Bu Ali Sina, Hamadan, Iran; 3 Department of Pharmacy/Chemistry, School of Life Sciences, University of Sussex, Falmer, Brighton, BN1 9QJ UK; 4 Drug Applied Research Center and Faculty of Pharmacy, Tabriz University of Medical Sciences, Tabriz, Iran

**Keywords:** Stability, Polyox matrices, Drug release, Drug solubility, Controlled release

## Abstract

Hydrophilic matrix systems are currently some of the most widely used drug delivery systems for controlled-release oral dosage forms. Amongst a variety of polymers, polyethylene oxide (PEO) is considered an important material used in pharmaceutical formulations. As PEO is sensitive to thermal oxidation, it is susceptible to free radical oxidative attack. The aim of this study was to investigate the stability of PEO based formulations containing different model drugs with different water solubility, namely propranolol HCl, theophylline and zonisamide. Both polyox matrices 750 and 303 grade were used as model carriers for the manufacture of tablets stored at 40 °C. The results of the present study suggest that the drug release from the matrix was affected by the length of storage conditions, solubility of drugs and the molecular weight of the polymers. Generally, increased drug release rates were prevalent in soluble drug formulations (propranolol) when stored at the elevated temperature (40 °C). In contrast, it was not observed with semi soluble (theophylline) and poorly soluble (zonisamide) drugs especially when formulated with PEO 303 polymer. This indicates that the main parameters controlling the drug release from fresh polyox matrices are the solubility of the drug in the dissolution medium and the molecular weight of the polymer. DSC traces indicated that that there was a big difference in the enthalpy and melting points of fresh and aged PEO samples containing propranolol, whereas the melting point of the aged polyox samples containing theophylline and zonisamide was unaffected.

**Electronic supplementary material:**

The online version of this article (doi:10.1007/s13346-016-0344-5) contains supplementary material, which is available to authorized users.

**Graphical abstract:**
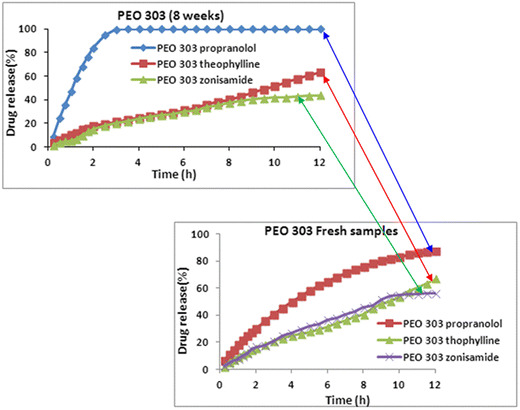
ᅟ

## Introduction

Controlled release (CR) deals with dosage forms intended to provide a therapeutic amount of drug to a specific site or location at the desired rate [1]. In the recent years, CR systems have gained much attention and have become an increasingly important strategy in therapeutic treatment. The underlying reason is that the CR systems allow the pharmacological effect to be maintained by releasing a drug to the desired target site at a controlled rate for an appropriate extended time. CR formulations have several potential advantages over conventional dosage forms, such as reducing high total dose, reducing dosing frequency and gastrointestinal (GIT) side effects as well as improving patient acceptance and compliances [2]. Advances in research aiming towards fundamental principles to bring both commercial and therapeutic values to health care products have led to the introduction of a new concept termed as ‘chronotherapy’. It refers to a clinical practice of synchronizing drug delivery in a manner consistent with the body’s circadian rhythm including disease states, to produce maximum health benefit and minimum harmful effects [3].

Hydrophilic matrix tablets are among the most popular delivery systems for oral controlled-release dosage forms. These hydrophilic matrices are widely accepted because of their biopharmaceutical and pharmacokinetics advantages over conventional dosage forms [4]. A hydrophilic matrix tablet generally consists of a mixture of drug, polymer and excipients such as filler or diluent as well as other excipients mainly used in common tableting process [5]. Hydrophilic matrix systems are simple to formulate, inexpensive and easy to produce and generally have a good in vitro and in vivo correlation [6]. From the wide choice of possible matrix materials, polyethylene oxide (PEO) has been used most frequently in the formulation of CR monolithic matrix tablets because of its hydrophilic gel-forming property, non-toxicity and cost effectiveness, insensitivity to the pH of the biological medium and ease of production [7, 8]. Its good compressibility also allows the preparation of hydrogel matrices by direct compression [9]. Polyox consists of a family of high molecular weight polyethers with molecular weights ranging from 100,000 to 7,000,000. These materials have many uses in the pharmaceutical industry, for instance, as dispersants, binders, viscosifiers, mucoadhesives and hydrophilic matrix tablets [10, 11]. In many of these applications, the properties that polyox imparts are dependent upon its molecular weight where both swelling and dissolutions are faster in the case of the lower molecular weight forms, which results in higher release rates. On the other hand, the extent of swelling is higher for higher molecular weights, and this is the dominant factor in drug release [12]. Water diffusion into the matrix results in a destruction of the polymer network. Once the macromolecules are disentangled, the polymer chains diffuse through the unstirred layer surrounding the tablet, which is characterized by a distinct polymer concentration gradient. With increasing molecular weight, the degree of entanglement of the macromolecules increases and vice versa [13]. When matrices containing PEO polymer come into contact with water, forces of attraction chiefly hydrogen bonding, start acting between polymer and water and the forces holding the polymer segments together are therefore reduced, and the polymer chains can swell consequently forming a gel layer at the matrix surface. Water-soluble drugs are mainly released by diffusion of dissolved drug molecules across the gel layer, while poorly water-soluble drugs are more likely to be released through erosion of the gel [14]. The controlling mechanism of drug release from PEO tablets is dependent upon the drug solubility, drug loading, the addition of water-soluble excipients and the molecular weight of the PEOs [15].

As PEO’s can undergo chain cleavage via auto-oxidation [16], the aim of this present study is to investigate the influence of accelerated storage conditions (at 40 °C over 8 weeks) on release rate of various drugs, with different solubilities, namely propranolol HCl, theophylline and zonisamide from two different molecular weight polyethylene oxides (750, and 303).

## Materials and methods

### Materials

PEO grades Sentry Polyox WSR 750 (ID166255), MW = 3 × 10^5^ and PEO 303 Sentry Polyox WSR 303 (ID166227) MW = 7 × 10^6^ manufactured by Dow chemical (Philadelphia, USA) and distributed by Colorcon (Kent, UK) were used. Propranolol HCl, theophylline and zonisamide were supplied by ELAN drug technologies (UK). Magnesium Stearate (MgSt) (Fischer Scientific) was required for its lubricating properties in preventing the produced tablet from sticking to the die walls and punch faces. All solvents used were of analytical grades and used as received.

### Tablet preparation

Direct compression method was used to ensure less variability in the final composition of the tablets. Tablet matrices were made at ratio of 1:1 drug/polymer (*w*/*w*) and with the total weight of 240 mg. To obtain a well-mixed composition, binary physical mixtures were blended in a TURBULA TF2 (Basel, Switzerland) machine for 10 min, prior to the direct compression using Enerpac tableting machine procured from Lobe Pharma with an 8-mm dye and punch at 1500 psi pressure. The dye and the punch were lubricated with magnesium stearate. Once prepared, all tablets were kept at room temperature. For stability test purposes, three to four tablets were kept at accelerated temperature 40 °C at time intervals of 2, 4 and 8 weeks in the oven in a closed glass container.

### Differential scanning calorimetry

Differential scanning calorimetry (DSC) was used to evaluate thermal properties and melting point of the polymer, drugs and the formulations with a Mettler Toledo DSC1 tester. The equipment was calibrated using indium and zinc at a scanning rate of 10 °C/min in aluminium pans under nitrogen gas. Approximately, 4–5 mg of sample was weighed and heated at a range between 25 and 260 °C at a heating rate of 10 °C/min. All samples were run in duplicates.

### Determination of the tablet hardness

In order to measure the force needed to fracture the tablet, at least three tablets were tested using tablet hardness tester 8 M Dr. Schleuniger obtained from JB Pharmatron Ltd. The average values of the tablet hardness were also determined.

### Dissolution parameters

Dissolution efficiency (DE_720min_) and mean dissolution rate (MDR) were determined to represent the dissolution rate from various preparations. The dissolution efficiency (DE) of pharmaceutical dosage forms is defined as the area under the dissolution curve up to a certain time, *t*, expressed as percentage of the area of the rectangle described by 100% dissolution in the same time. DE is described below by Eq. (1) [17]:

1 $$ DE=\frac{{\displaystyle \underset{0}{\overset{t}{\int }}y\times dt}}{y_{100}\times t}\times 100\% $$

where *y* is the percent drug release as the function of time, *t*, *t* is the total time of drug release and *y*100 is 100% drug release. An alternative parameter that describes the dissolution rate is the mean dissolution time (MDT); the most likely time for a molecule to be dissolved from a solid dosage form. Therefore, MDT is the mean time for the drug to dissolve under in vitro dissolution conditions. This is calculated using the following Eq. (2) [17].

2 $$ MDT=\frac{{\displaystyle \sum_{j=1}^n{t}_j\;\varDelta\;{M}_j}}{{\displaystyle \sum_{j=1}^n\varDelta\;{M}_j}} $$

where *j* is the sample number, *t*_*j*_ is the midpoint of the *j*th time period (easily calculated with [*t* + (*t* − 1)]/2 and Δ*M*_*j*_ is the additional amount of drug dissolved between *t*_*j*_ and *t*–1. Microsoft Excel was then used to calculate the cumulative percentages of DE and MDT.

### Dissolution test

Dissolution test was carried out in an ERWEKA DT 700 type dissolution tester at 37 °C ± 0.1 °C. The USP paddle method [18] was used to monitor the release profiles of the tablets. The dissolution medium was 900-ml deionised water and the paddles rotated at 100 rpm. The samples were withdrawn and analysed at every 15 min for up to 2 h, and thereafter, every 30 min up to 12 h. The concentration of the propranolol HCl, theophylline and zonisamide was determined with a uv-vis spectrophotometer (UV-1700 pharmaspec) provided by SHIMADZU at 290, 271 and 270 nm, respectively.

### X-ray powder diffraction studies

The X-ray power diffraction (XRPD) patterns of PEO loaded with various drugs at different storage time were determined using a Siemens Diffractometer (Siemens, D5000, Germany) with CuKa radiation (1.5405 Å). The instrument was calibrated using a silicon standard. The tube voltage and amperage were set at 40 KV and 40 MA. The samples were analysed between 2θ angles of 2 and 35° with a step size of 0.02° at 2 step/s.

### Scanning electron microscope analysis

Electron micrographs of PEO matrices were obtained using a scanning electron microscope (SEM) (Leica Cambridge S360, UK) operating at 15 kV. The samples were mounted on a metal stub with double-sided adhesive tape and coated under vacuum with gold in an argon atmosphere prior to observation. Micrographs with different magnifications were recorded to study the morphology of the solid dispersions.

### Gel permission chromatography

Gel permeation chromatography (GPC) was used to study the stability of the polymer by measuring its weight average molecular weight. A method similar to that described by Crowley et al. [20] was used where all standards and samples were prepared at 0.5% *w*/*w* concentrations in mobile phase under gentle agitation for 10 h prior to injection. A PL-GPC 50 Model (Varian, UK) was used with a PL-aquagel-OH 8 μm column (Agilent, UK). Samples were compared against standards (EasiVial®, Agilent, UK) to look at qualitative changes in molecular weight. The mobile phase was double-distilled HPLC water (Fisher UK) with 0.1 M sodium nitrate and was pumped at a flow rate of 0.8 ml/min. The column was held at an operating temperature of 35 °C during the analysis.

### Viscosity study

Ground tablet samples were prepared at a 0.5% *w*/*v* concentration in distilled water using gentle agitation on a radial shaker for 12 h at 25 °C. They were tested using a Brookfield Model DV-II+ Pro viscometer (Harlow, UK) using spindles 61 and 62 together with rotation speeds of 50 and 100 rpm as required; results presented are an average of three runs.

### Similarity factor (f2) measurement

In order to determine the similarity between the obtained drug release profiles, the *f*_2_ was calculated according to Eq. (3) below:

3 $$ {f}_2=50 \log \left\{{\left[1+1/\ {\left(Rt\hbox{--} Tt\ \right)}^2\right]}^{-0.5}\times 100\ \right\} $$

This being a mathematical treatment of the dissolution data where *n* is the number of test points for the samples, *w*_*t*_ is the optional weight factor, *R*_*t*_ is the reference assay at time point *t* and *T*_*t*_ is the test assay at time point *t*. An *f*_*2*_ value between 50 and 100 suggests a similarity between the two release profiles and the closer the value is to 100 the more similar or identical the profiles are. Also, dissimalrity occurs with decreasing values less than 50.

## Results and discussion

### The influence of storage conditions on true density and tablet hardness

All formulations were relatively robust in terms of true density. The results demonstrated that there was no significant difference (*p* > 0.05) between true density of PEO polymer formulations before and after storage times even when stored for 8 weeks at accelerated temperature condition (40 °C). The range of true density for fresh samples was between 1.22 and 1.25 g/cm^3^ and after 8 weeks, the true density was almost the same (1.21–1.24 g/cm^3^) with just a very nominal change of about 0.8–0.9%. In order to analyse the moisture content, a Loss on Drying (LoD) test was performed to a constant weight at temperature 10 °C below the melting point of PEO. LoD analysis to elucidate the moisture content of the powder obtained from the crashed tablets showed a very nominal change in mass being about ∼1% *w*/*w* without any significant effect on the tablets (e.g. density). It is apparent that PEO molecular weight plays an influential role in determining the tablet hardness. The effect of storage conditions on the tablet hardness of various drugs with a ratio of drug**/**polymer (1:1) is shown in Table 1. Looking at the hardness of different propranolol-polyox (soluble drug) grades at time zero (fresh tablets), there is a very modest increase of tablet hardness as polymer chain length increased (Table 1). This could be due to a better compactibility of PEO high molecular weight compared to that of lower molecular weight (Table 1). The table also showed that there was a decrease in the hardness of the tablets when storage time progressed. In other words, generally, the longer the storage time the lower the hardness. This might be due to degradation, depolymerisation and crystallinity changes of the aged PEO samples. Our results correlated with previous research carried out by Engineer et al. (2004), who investigated the effect of temperature on the hardness of sustained-release diphenhydramine HCl tablets [19].

**Table 1:** Effect of storage time on polyox tablet containing different drugs hardness stored at 40 °C

PEO/drug	Time (week)	Hardness ratio 1:1
750/propranolol	Fresh	93.1 ± 0.5
750	2 weeks	88.0 ± 0.5
750	4 weeks	84.0 ± 0.2
750	8 weeks	79.2 ± 0.1
303/propranolol	Fresh	100.3 ± 0.1
303	2 weeks	94.5 ± 1.0
303	4 weeks	89.0 ± 0.5
303	8 weeks	84.1 ± 0.3
750/theophylline	Fresh	95.5 ± 1.0
750	2 weeks	93.9 ± 0.5
750	4 weeks	91.5 ± 0.4
750	8 weeks	86.1 ± 0.1
303/theophylline	Fresh	105.0 ± 0.5
303	2 weeks	104.5 ± 0.2
303	4 weeks	104.2 ± 0.4
303	8 weeks	104.0 ± 1.0
750/zonisamide	Fresh	96.0 ± 0.5
750	2 weeks	98.1 ± 0.3
750	4 weeks	99.5 ± 0.3
750	8 weeks	102.0 ± 0.4
303/zonisamide	Fresh	108.0 ± 1.0
303	2 weeks	114.0 ± 0.5
303	4 weeks	122.0 ± 2.0
303	8 weeks	133.0 ± 1.0

These results (Table 1) in addition shows the summary of the hardness measured before and after storage times for theophylline tablet matrices. It can be seen in Table 1 the measured values for batches containing PEO 750 were higher at time 0 compared to aged tablets (2, 4 and 8 weeks). But all tablets containing PEO 303-theophylline showed no significant difference, as the hardness of the tablets were almost the same before and after the storage (2, 4 and 8 weeks). For example, tablet hardness of PEO 750 before and after storage time was 95.5 and 86.1 N, respectively, while the range of tablet hardness for PEO 303 was between 105 and 104 N.

Moreover, the results obtained for zonisamide tablet matrices differed significantly compared to the polyox matrices containing soluble and semi soluble drugs. It can be seen in Table 1 that the average hardness of PEO 303 tablets stored for 0, 2, 4 and 8 weeks is 108, 114, 122 and 133 N, respectively, whereas in the case of PEO 750, the hardness of the tablets increased from 96 to 102 N when the storage time increased from 0 to 8 weeks. It is apparent that PEO molecular weight and solubility of zonisamide play an important role in the tablet hardness, as a significant increase (ANOVA test *p* < 0.05) in tablet hardness was observed for high molecular weight PEO 303, while there was only a slight increase in the hardness of tablets when low molecular weight PEO 750 polymer was used.

### The influence of storage condition and various molecular weights of polyox on propranolol HCl release

**Fig. 1:**
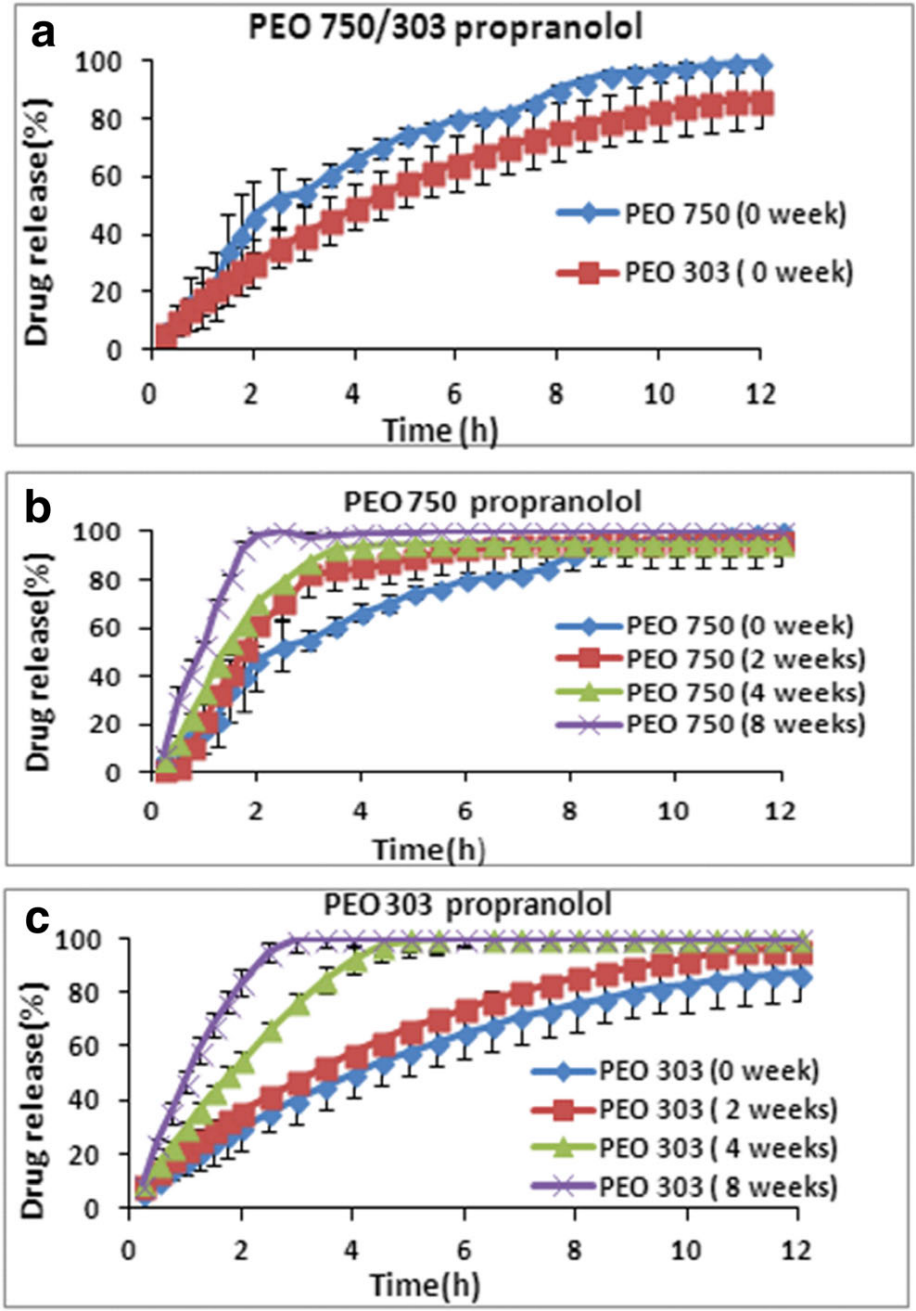
The effect of storage time and various molecular weights on drug release of propranolol at (1:1) drug/polymer ratio and stored at 40 °C. **a** PEO 750/303 effect of MV. **b** PEO 750. **c** PEO 303

**Fig. 2:**
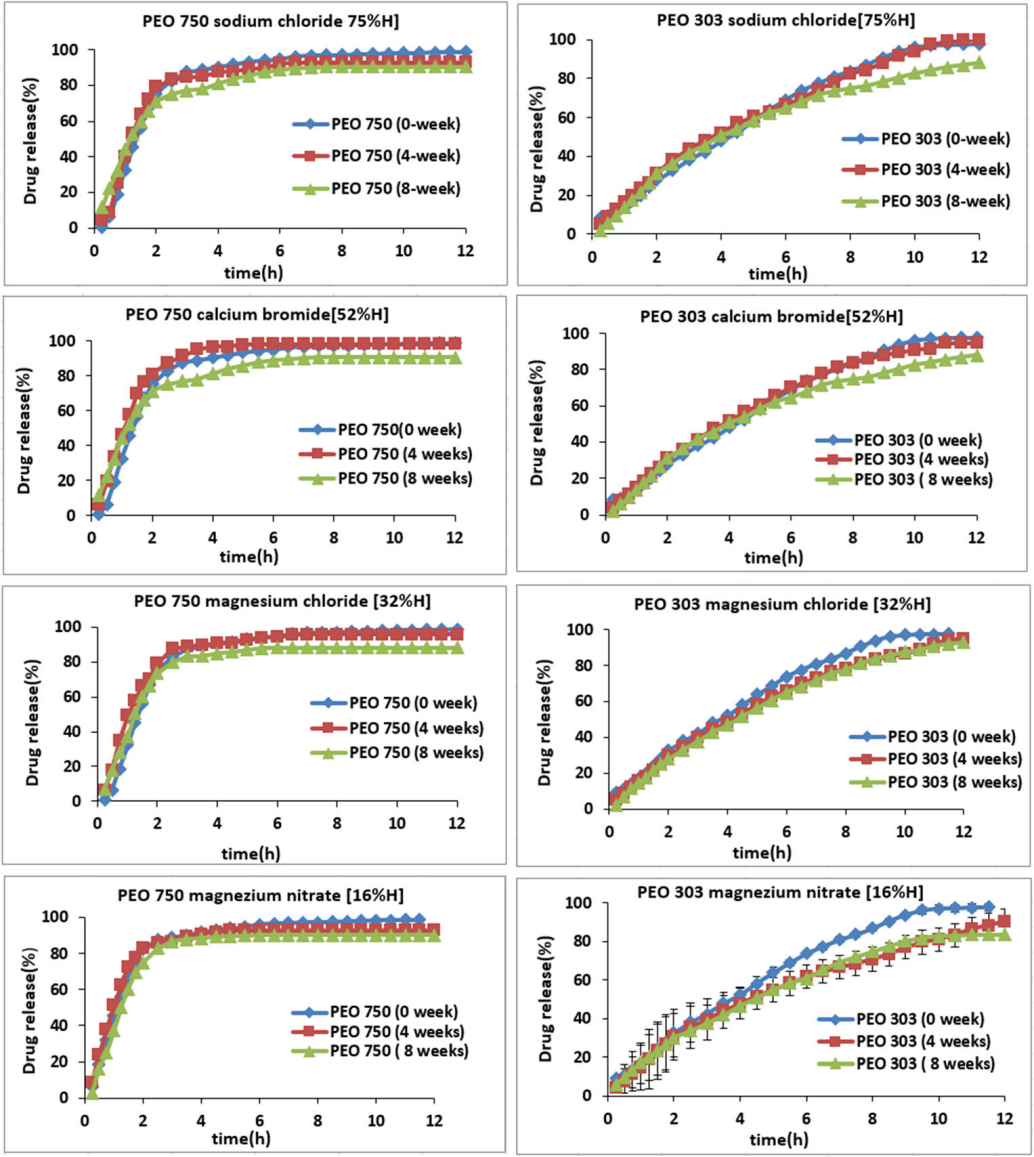
The effect of different relative humidity on the drug release of propranolol at 16–75% RH on storage

In order to investigate the effects of storage conditions and different molecular weight PEOs on drug release rate of propranolol HCl, dissolution test was conducted and the dissolution profiles are shown in Fig. 1. Polymer molecular weight is one of the primary parameters that dictate drug release rate and dissolution from solid dosage forms, and it influences the in vivo bioavailability and therapeutic efficacy [20]. Figure 1a summarizes the effect of molecular weight on drug release, with low molecular weight PEO 750 achieving faster drug release than high molecular weight PEO 303. Drug release from high molecular weight PEO is principally governed by the material swelling and diffusion rather than by polymer erosion, and progressive decline of the drug’s diffusive conductance in the growing swollen layer, making the stronger gel layer less liable to erosion. Conversely, drug release from low molecular weight PEO predominantly involves swelling and erosion, in which the synchronization of these may lead to release kinetics. The results also showed that lower molecular weight PEO 750 exhibited about 50% of drug release in less than 2 h, while in case of PEO 303 almost 60% of drug release occurred within 6 h. It is well known, for a water-soluble polymer, the higher the molecular weight the higher the viscosity of the polymer solution and the slower the swelling and dissolution rate [21]. The results showed that there was a gradual release, followed by a progressive increase in drug release with the higher molecular weight polyox. For soluble drugs like propranolol (solubility of propranolol HCl is 50.0 mg/ml in water), the swelling of the polymer is due to the penetration of the medium into the matrix system that elicits a displacement of the solid particles of the drug in the gel layer [22, 23]. In general, the solubility of the drug may govern the type of the polymer to be used, and the need to formulate with polymer mixtures, and the incorporation of co-excipients [23]. Figure 1b, c shows that the storage time also had an impact on propranolol HCl release from polyox 750 or 303. The drug release was faster at longer storage times and it can be ranked as 8 weeks >4 weeks >2 weeks >0 week. Upon accelerated storage conditions, it is apparent that there is a shortening in polymer chain length, suggesting the occurrence of PEO oxidation, thus instability causing faster drug release with time [24]. On the basis of the above information, some of the features in the dissolution rate of PEO can be confirmed particularly by similarity factor *(f2)* values. All *f2* values for PEO 750 and 303 (lower and higher molecular weights) are reported to be less than 50 when the fresh tablets were compared with the aged polyox tablets containing propranolol which is an indication of no similarity between their release profiles (Table 2). The effect of humidity condition during the storage did not have significant impact on the release profiles of propranolol HCl from the PEO matrices. As can be seen in Fig. 2 that while changing the relative humidity during the storage of the samples from 16% to very high 75% RH values, the release profiles for both grades remained almost the same. However, a slight increase in the release profile of aged PEO 303 samples (4 and 8 weeks) was observed when the humidity was increased from 16% to higher values. This could possibly be attributed to the fast material swelling and diffusion on high relative humidity condition and hence the faster release of water-soluble propranolol.

**Table 2:** Similarity values *f*_*2*_ for propranolol, theophylline and zonisamide PEO tablet release profiles at different storage times compared to controls at time zero for (1:1) ratio

Polyox/drugs	2 weeks	4 weeks	8 weeks
750 (propranolol)	45.5	21.3	20.6
303	23.7	27.4	12.4
750 (theophylline)	50.5	42.2	32.1
303	76.3	63.5	70.2
750 (zonisamide)	64.3	59.6	55.1
303	65.1	73.6	56.6

Dissolution efficiency (DE) and mean dissolution time (MDT) were used to compare the dissolution data as described by Khan (1975) [17]. The results of fitting the dissolution data with the dissolution criteria are shown in Table 3. Dissolution efficiency values are consistent with dissolution profiles and these data confirmed that the drug release rate from PEO 750 as lower molecular weight is faster when they are stored at 40 °C for different times compared to the fresh tablets. For instance, the dissolution efficiency value of PEO 750 at time 0 was 62% whereas this value increased to 92% for tablet matrices stored for 8 weeks at 40 °C. A similar pattern was obtained for the higher molecular weight PEO 303 (Table 3). The results obtained for MDT confirmed the same trend (with DE) for both low and high molecular weight PEO. For instance, MDT for fresh PEO 750 tablets was 2.59 h while this value decreased to 0.96 h at 8-week storage time which is an indication of very fast drug release for the tablets stored at 40 °C (Table 3).

**Table 3:** Effect of storage time on dissolution parameters of propranolol PEO tablet matrices

PEO	Time (weeks)	DE (%)	MDT(h)
750	0	75.0 ± 2.81	2.59 ± 1.24
750	2	84.0 ± 4.0	1.81 ± 0.26
750	4	88.0 ± 0.83	1.48 ± 0.04
750	8	92.0 ± 0.26	0.96 ± 0.03
303	0	60.0 ± 4.06	3.93 ± 0.07
303	2	68.0 ± 1.68	3.72 ± 0.03
303	4	85.0 ± 1.15	1.93 ± 0.02
303	8	91.0 ± 0.93	1.16 ± 0.01

*DE* dissolution efficiency, *MDT* mean dissolution time

**Fig. 3:**
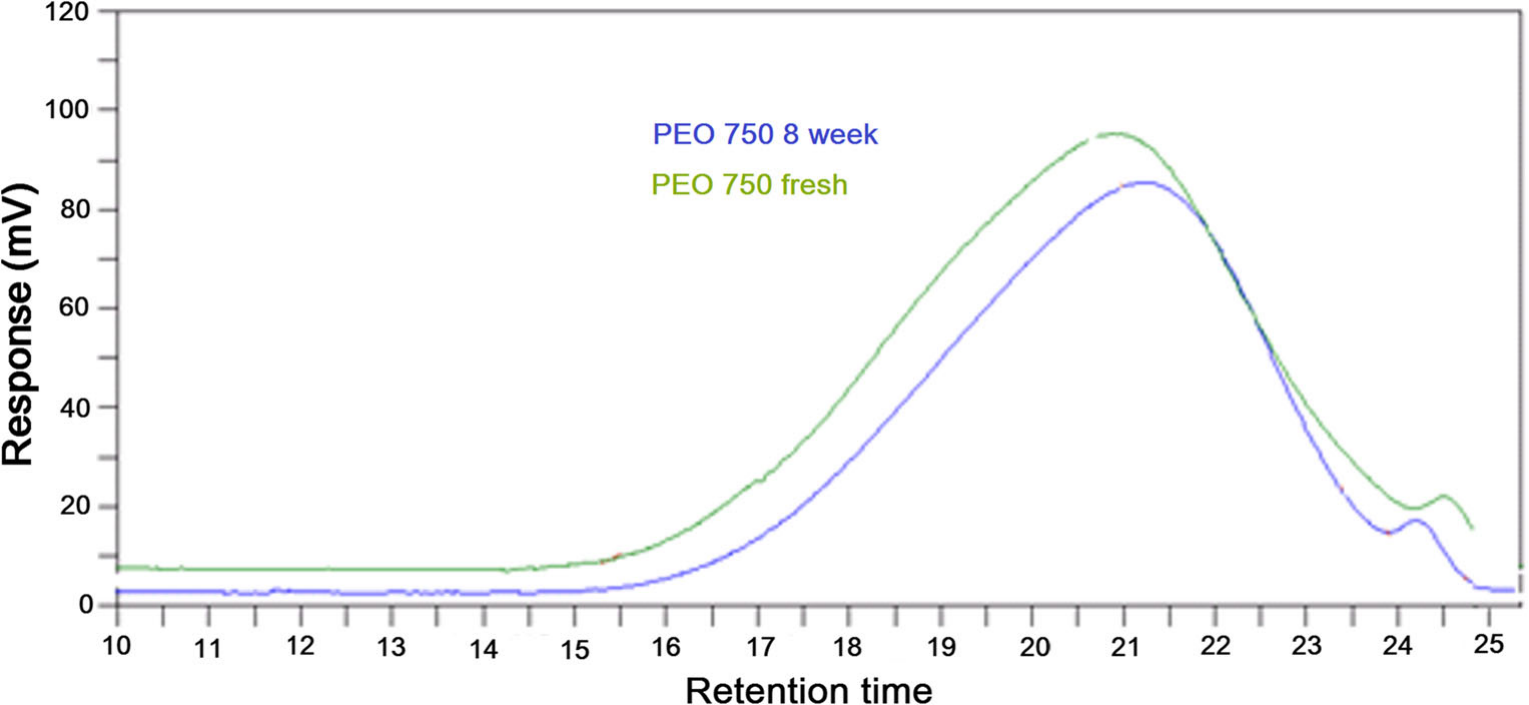
GPC profiles for PEO 750 fresh compared to 8-week samples stored at 40 °C

The changing in molecular weight usually measure by GPC. Figure 3 shows GPC profile for lower molecular weight PEO 750. As can be seen from Fig. 3 that the sample stored at elevated temperature (40 °C) over 8 weeks indicated a significant shift to longer retention times as results of ageing showing a noteworthy decline in the molecular weight of the aged sample. During storage, PEO can degrade to lower molecular weight with shorter chain length and lower solution viscosity. Its physical appearance becomes soft, waxy with a distinctive smell [20]. Our results correlated with previous research investigating the effect of storage time on the molecular weight of sustained-release tablets [20, 25].

Another approach to confirm the effect of storage time on PEO samples is viscosity testing which is done by previous researcher to revise the poverty and degradation of Polyox® [26, 27]. Our data are in good correlation with the previous researchers who stated that a significant decreasing in molecular weight of PEO was occurred when the PEO samples stored at elevated temperature. Viscosity data (Table 4) clearly shows that there is major reduction in viscosity of fresh samples when they were stored at elevated temperature at 40 °C for 8 weeks. For instance, fresh PEO 750 showed a viscosity of 60 (cp), while it reduced to 52 (cp) at 8-week storage. Similar pattern was obtained for higher molecular weight PEO 303 (Table 4).

**Table 4:** Viscosity data for ground tablet samples containing PEO 750 and 303 at time zero and 8 weeks storage at 40 °C

PEO	PEO 750/viscosity (cp)	PEO 303/viscosity (cp)
Fresh	60	736
8 weeks	52	109

### The influence of storage time and various molecular weights of PEOs on theophylline release

**Fig. 4:**
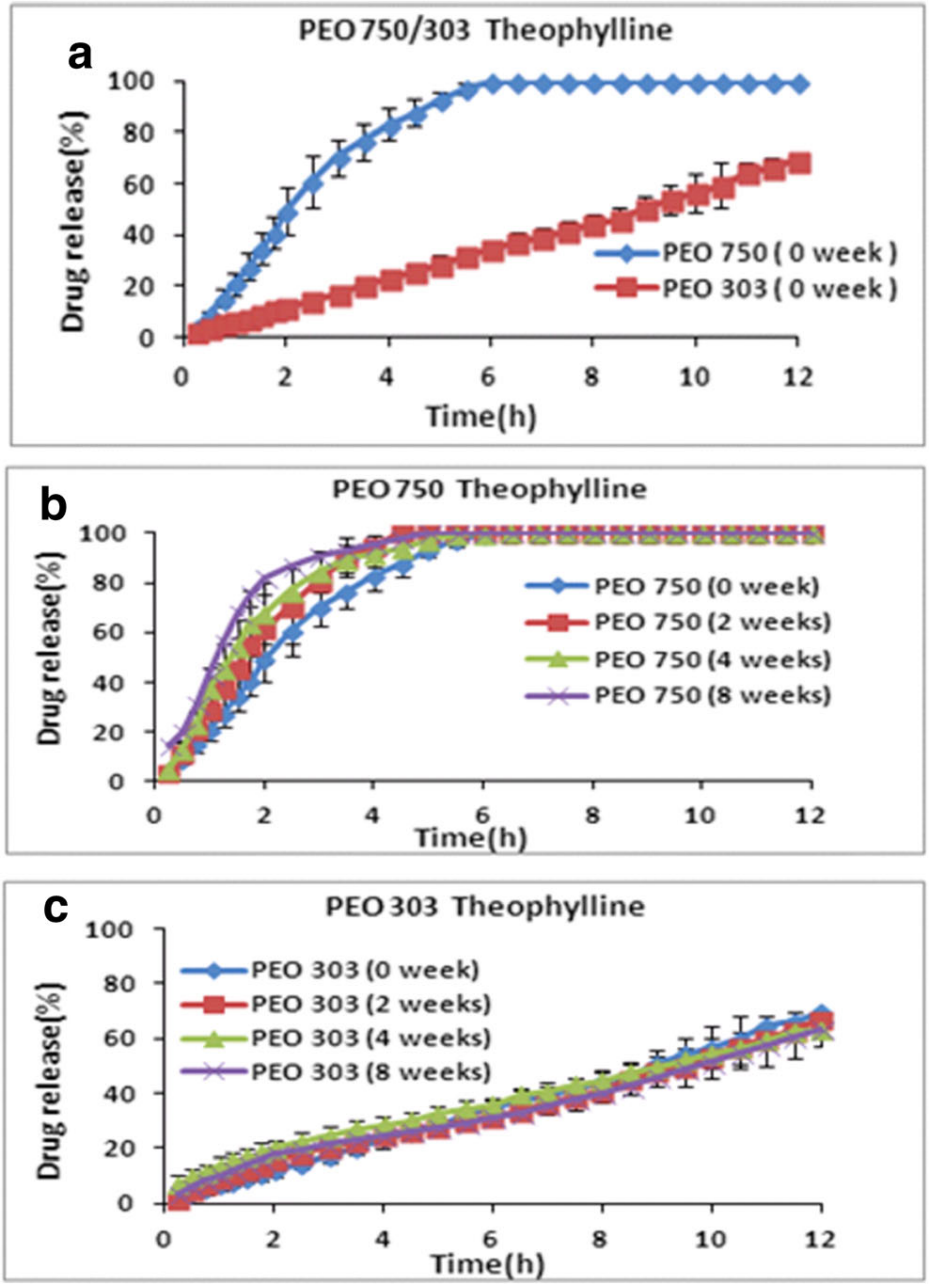
The effect of storage time and various molecular weights on drug release of theophylline at (1:1) drug/polymer ratio and stored at 40 °C. **a** PEO 750/303 effect of molecular weight. **b** PEO 750. **c** PEO 303

Theophylline with less solubility (8 mg/ml) compared to propranolol HCl was selected as another model drug with moderate solubility to investigate the effect of ageing on the drug release from polyox 750 and 303 matrices. The release profiles of theophylline from fresh polyox samples are presented in Fig. 4a. The results showed that, once again, the drug release was faster from low molecular weight PEO 750 and slower from high molecular weight PEO 303. This suggests that the higher molecular weight of aged PEO matrices have the ability to swell and form stronger gel upon hydration which is less likely to erode. These findings are in good agreement with the results obtained in various studies [26–28]. The same conclusion was previously drawn for highly water-soluble drug. The reason for this pattern has been presented earlier in the case of propranolol HCl matrices [26–28].

As expected, comparison of the dissolution profiles of fresh matrices containing two different drugs so far showed that the drug release is slower from the matrices containing theophylline (Fig. 4a) compared to polyox matrices containing propranolol HCl (Fig. 4a). This could be attributed to the less solubility of theophylline in the dissolution medium compared to propranolol HCl compared to the release profiles of aged polyox samples containing highly water-soluble drugs particularly in case of polyox 303.

It is interesting to note that the effect of storage time on theophylline release was different compared to the release profiles of aged polyox samples containing highly water-soluble drugs particularly in case of polyox 303. In the case of polyox 750, the drug release became faster at longer storage times (8 > 4 > 2 > 0 weeks (Fig. 4b). In contrast, Fig. 4c shows no significant differences between the fresh and the aged tablets (8 weeks) when polyox 303 was used. It is apparent from the figure that all release profiles were super imposable with no significant changes in the drug release. This is supported by Bigger et al. (1991), who investigated the effects of molecular weight and thermal oxidation on the dynamic mechanical response of polyethylene oxide [28]. Comparing the release profiles of water-soluble drug diltiazem with theophylline from polyox 750 showed that the gap between the release profiles of fresh samples and aged samples is much smaller for theophylline (Fig. 4b) compared to matrices containing highly water-soluble drugs (Fig. 1b).

This further investigated in separate studies that correlate our data with respect to PEO stability. The increase in drug release rate observed varies with molecular weight [29, 30]. They observed that the partly soluble drug theophylline tend to follow diffusion/erosion release mechanism depending on the grades of polymer used. From their data, the mechanism of theophylline release from the higher molecular weight matrices was by diffusion and from the lower molecular weight by erosion.

To confirm the above findings, the similarity factor (*f2*) was calculated and data are presented in Table 2 *.* This table shows that all *f2* values for polyox 750 are less than 50 except tablets stored for 2 weeks which is an indication of no similarity between fresh sample and aged polyox formulations (4 and 8 weeks). In the case of polyox 303, all *f2* values were greater than 50 indicating similarity between fresh and aged formulations. All these *f2* values supported the conclusion drawn for release profiles of fresh and aged polyox matrices containing theophylline. Dissolution efficiency (DE) and mean dissolution time (MDT) were also used to compare the dissolution data as described by Khan (1975). The results of fitting the dissolution data with the dissolution criteria are shown in Supp. Table 1 *.* Dissolution efficiency values are consistent with dissolution profiles and these data confirmed that the drug release rate from PEO 750 is faster when they are stored at 40 °C for different times. For instance, the dissolution efficiency value of PEO 750 at time 0 was 80.0% whereas this value increased to 90.0% for aged matrices stored for 8 weeks at 40 °C.

As can be seen from Supp. Table 1, almost similar DE values were obtained for fresh polyox 303 matrices (34.0%) and aged matrices for 8 weeks (33.0%). The results obtained for MDT confirmed the same conclusion mentioned above as MDT for fresh PEO 750 tablets was 2.35 h while this value decreased to 1.36 h at 8 weeks storage time which is an indication of faster drug release for the tablets stored at 40 °C for 8 weeks. No significant changes (*p* > 0.05) were observed for MDT in the case of fresh and aged polyox 303 matrices.

### Effect of storage time and molecular weight of PEOs on zonisamide release

**Fig. 5:**
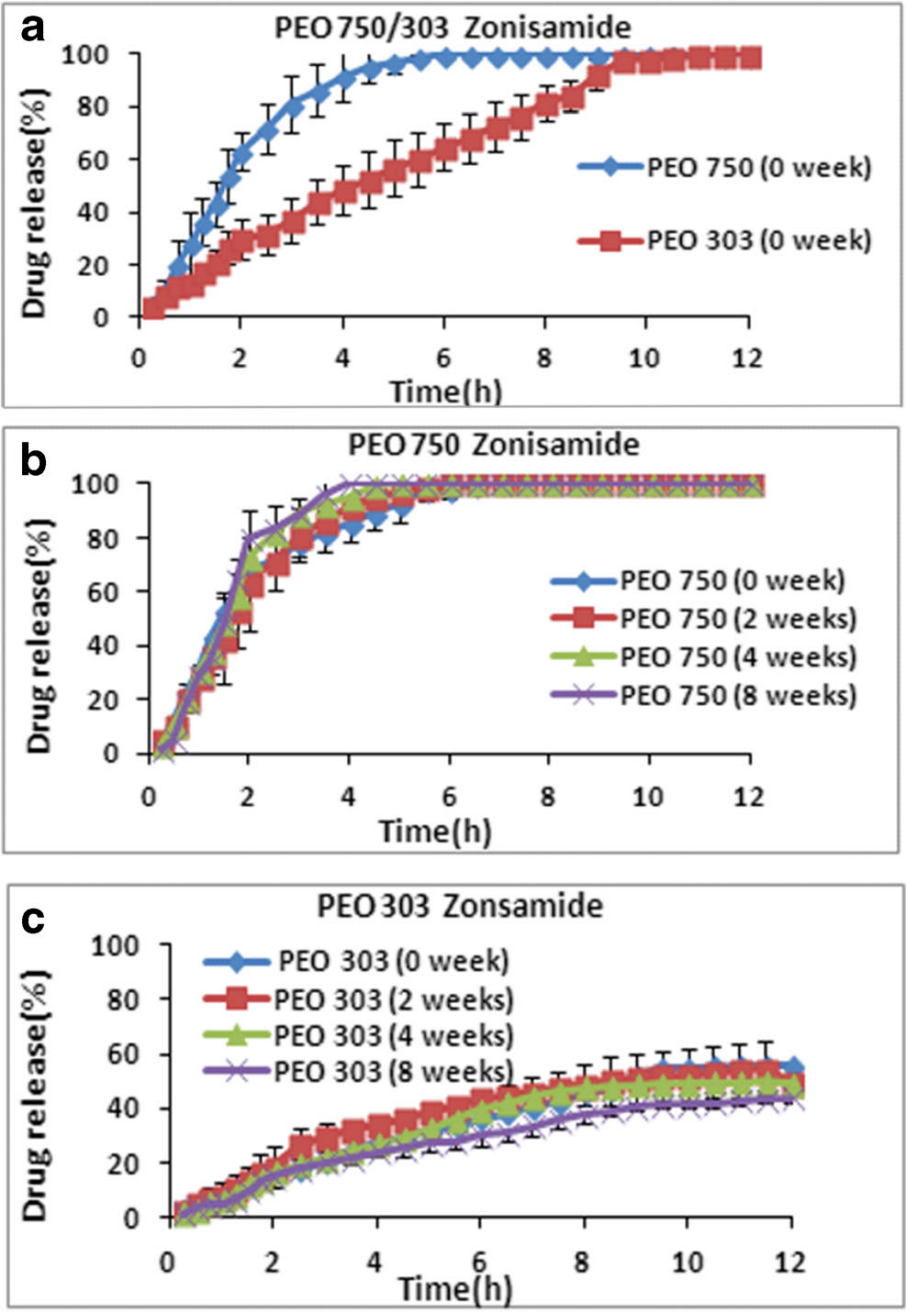
The effect of storage time and various molecular weights on drug release of zonisamide at (1:1) drug/polymer ratio and stored at 40 °C. **a** PEO 750/303 effect of molecular weight. **b** PEO 750. **c** PEO 303

Zonisamide was selected as a very poorly water-soluble drug (0.8 mg/ml) in the present study to cover the full range of solubility. In order to investigate the effects of storage conditions and different molecular weight PEOs on release rate of zonisamide, various PEO molecular weights (750 and 303) were chosen as the inert matrix and dissolution profiles are shown in Fig. 5. As expected again, the drug release was faster from low molecular weight of polyox compared to high molecular weights Fig. 5a. Thus, concluding that molecular weight has immense bearing upon the amount of drug released from tablets and the thermal oxidation of PEO being highly dependent upon this [20]. The reason for this release trend was discussed in detail earlier in this study. Figure 5b, c shows the drug release profiles of the two different molecular weight (303 and 750) PEO matrices containing zonisamide at different storage times of 0, 2, 4 and 8 weeks. As it can be seen from the graphs that in the 12-h dissolution run, the amount of drug released increases in both PEO 303 and 750; however, as expected, drug release from PEO 750 is faster compared to PEO 303. The dissolution test for PEO 750 showed almost 100% drug release within 5 h of the dissolution test for all storage times, whereas for PEO 303 even after 12 h of dissolution test, 100% drug release is not obtained and the graph continues to increase. This further confirmed that high molecular weight PEO has the potential to be used as the novel sustained-release carrier for the oral delivery of zonisamide. However, in order to achieve the therapeutic level of zonisamide, a lower molecular weight grade of PEO may be more suitable other than PEO 303 and/or longer period dissolution time, due to slower drug release over 12-h period of time as seen in this experiment. This is due to poor solubility of zonisamide in the dissolution medium. In terms of stability, for PEO 750, a slight difference in drug release could be observed after storing the samples under stress conditions. On the other hand, drug release from PEO 303 stored for the first 4 weeks is similar; however, it decreases significantly after 8 weeks. As no other excipients were added in these formulations, the only factor that could possibly affect the dissolution is the drug molecule itself. This suggests that not only the physical property of the polymer determines the release changes from the dosage form in the course of storage but also the properties of the drug molecules such as solubility may affect the release kinetics of PEO 303 matrices.

On the basis of above information, some of the features in the dissolution rate of PEO can be elaborated particularly by similarity factor *(f2)* values (Table 2). It is evident from Table 2 as all *f2* values for zonisamide with both PEO 750 and 303 are reported to be greater than 50 when the fresh tablets were compared to aged polymer formulation which is an indication of similarity between their release profiles. This suggests the changes in the drug release with storage time were not significant.

Dissolution efficiency (DE) and mean dissolution time (MDT) were used to compare the dissolution data and they are shown in Supp. Table 2 *.* Dissolution efficiency values are consistent with dissolution profiles and these data confirmed that the drug release rate from PEO 750 is slightly faster when they are stored at 40 °C for different times although, it was not significant. For instance, the dissolution efficiency value of PEO 750 at time 0 was 84.0% whereas this value only increased to 87.0% for aged matrices stored for 8 weeks at 40 °C which is an indication of no significant changes in aged polyox samples over 8-week storage time.

In contrast, as it can be seen from Supp. Table 2 for higher molecular weight PEO 303, there was slight decreasing in DE values during storage time which is indicated possibly no significant degradation was happened over 8-week storage time. For example, DE values for fresh and 8-week samples were 35.0 and 28.0%, respectively. The results obtained for MDT confirmed the same conclusion, as MDT for fresh PEO 303 tablets was 4.11 h while this value increased to 4.16 h at 8-week storage time which is an indication of a slight decrease in the dissolution rate as the time progressed.

All these results indicated that the drug release from aged polyox samples containing poorly water-soluble drugs are more stable than the aged polyox matrices containing highly water-soluble drug (propranolol HCl). This shows that when various drugs are formulated as controlled-release matrices using polyox powders, the solubility of drug and molecular weight of polyox should be taken into consideration to achieve a suitable approach should be adopted to a stable release profile for drugs with different solubility. Since zonisamide is a very poorly soluble drug and the ratio of polymer to drug used in the formulation is 1:1, it can be assumed that the hydration and swelling of the polymer are also affected by the drug molecule for which the result obtained is as expected. The effects of drug molecular interaction with various polymers were studied and can be found in literature but not for PEO. As PEO are a non-ionic material, interaction between drug and polymers is less likely to take place other than hydrogen bonding (H bond)**.** Study by Sarisuta et al. (1999) revealed that erythromycin molecule and non-ionic HPMC polymer chain would probably interact through hydrogen bonding [31]. Moreover, computer simulation by Kiss et al. (2008) showed that the interaction between theophylline and poly (ethylene oxide) is stronger and the energy gain of the H bond between these two molecules is greater than in the case of metronidazole. Thus, by using this phenomenon, it can be said that a possible interaction between the polymer and the drug molecule may have taken place and the new bond structure between them may perhaps prevent oxidation and have altered the dissolution. Additionally, the –OH end groups in PEO are capable of H-bond formation with water molecules [20]. Thus, the lower molecular weight PEO 750 hydrates faster which facilitates the penetration of the water molecule into the matrix due to the presence of more –OH groups. On the other hand, PEO 303 forms a much thicker gel, leaving less opportunity for water molecules to get imbibed and interact with drug molecules likewise because of this the drug release from PEO 303 is slower [32, 33]. Moreover, as explained earlier with time formulations containing PEO 303 required more strength to break the tablets (See Table 1). Thus, combining both the hardness and dissolution data, it can be predicted that the interaction between the polymer and the drug may have increased with storage time which lead to stronger gel formation and therefore significantly decreased drug release from PEO 303.

In other words, although the samples containing propranolol HCl showed a significant (*p* < 0.05) reduction in the enthalpies after 8 weeks compared to the fresh samples, the reduction in the enthalpies of fresh samples including poorly soluble drug such zonisamide is not as significant as aged samples. This indicates that special care must be taken when formulating controlled-release matrices containing highly water-soluble drugs such as propranolol with polyox polymers.

### SEM and XRPD study

**Fig. 6:**
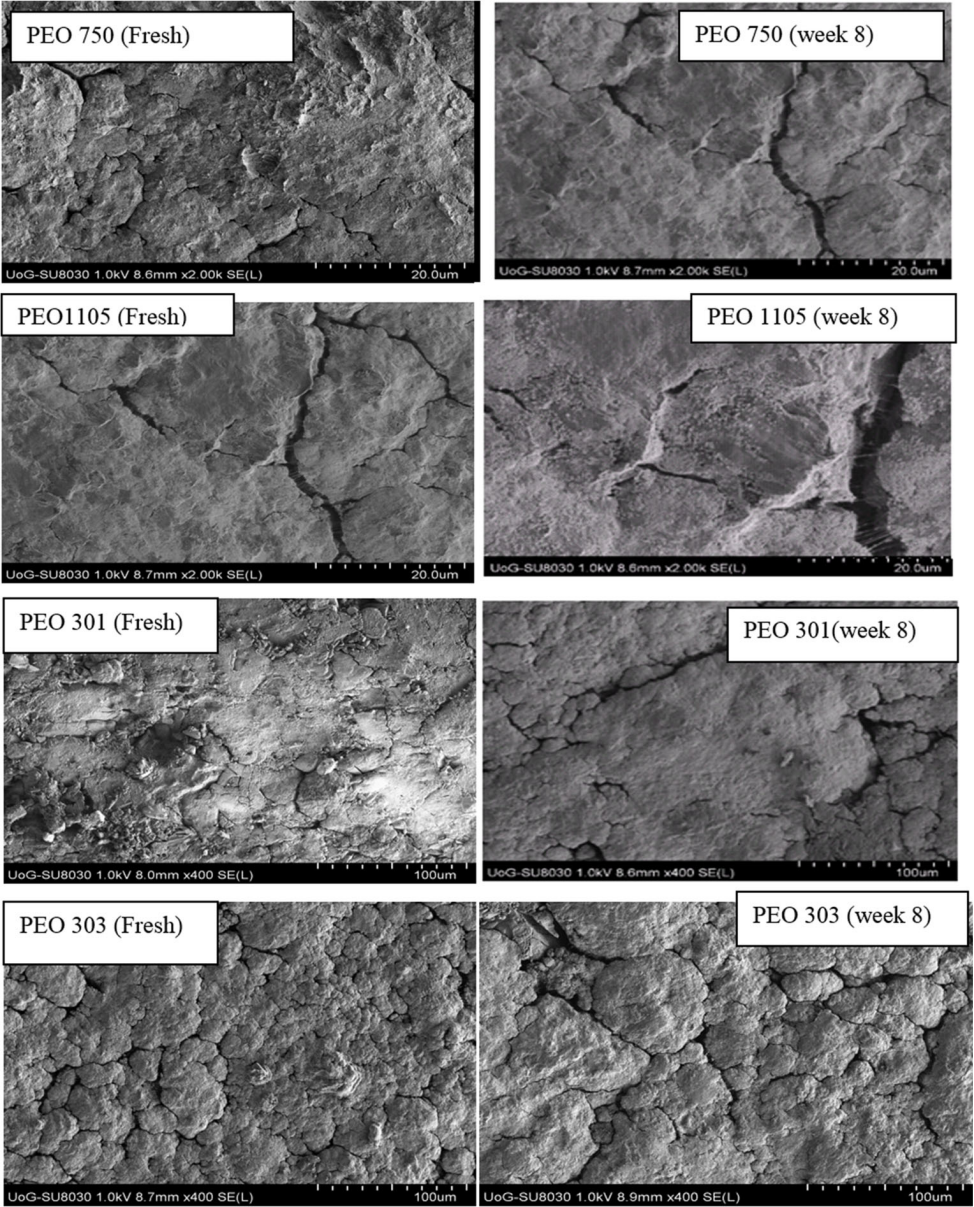
SEM of various molecular weight PEOs at zero and week 8 storage times that were obtained at 25 °C

SEM was used to find any changes in particle shape and morphology of PEO matrices before and after storage conditions when they were stored at elevated temperature at 40 °C for 8 weeks. The results of SEM for various molecular weights PEO are shown in Fig. 6. The figure shows there was no significant change in morphology of PEO matrices before and after the storage. This means that there was no significant evidence detected by SEM that could correlate the changes in the dug release with storage time.

**Fig. 7:**
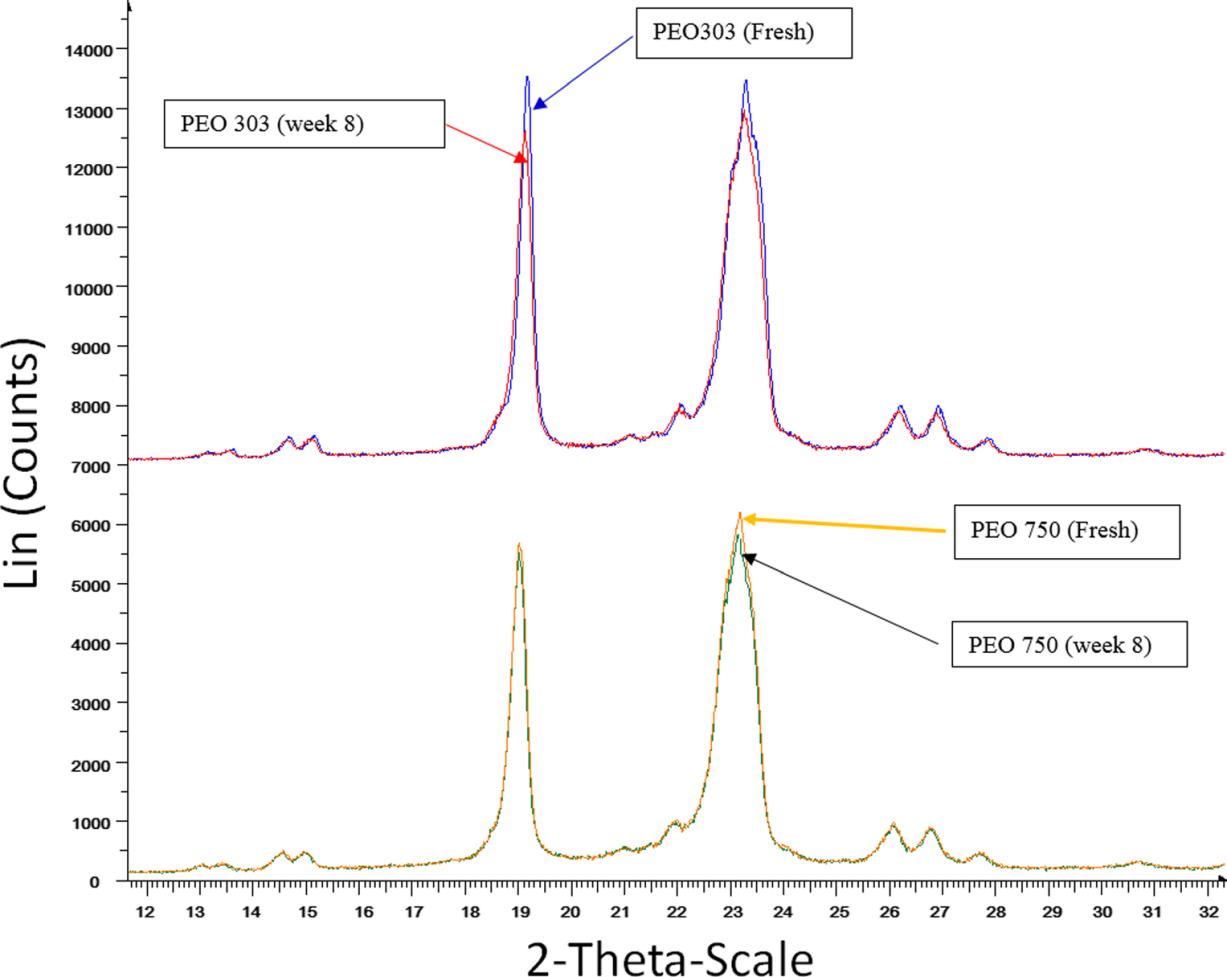
X-ray diffraction (XRD) patterns of various molecular weights PEO750 and 303 at zero and week 8 storage times

XRD was also used to see if any changes in the crystallinity of the samples could be found after storing them for 8 weeks at 40 °C. Figure 7 shows the XRD patterns of the different formulations with molecular weight PEO (750 and 303) before and after storage at 40 °C. XRPD results demonstrated that the solid state of PEO before and after storage time was similar. The only detectable difference between the fresh and aged polyox samples is the intensity of the peaks. The aged samples showed slightly smaller intensity compared to the fresh samples. This may be an indication of less crystallinity for the aged samples which might be responsible for faster drug release from aged polyox samples. This was also confirmed by DSC data in which aged samples showed lower enthalpies compared to the fresh samples.

### DSC analysis

**Fig. 8:**
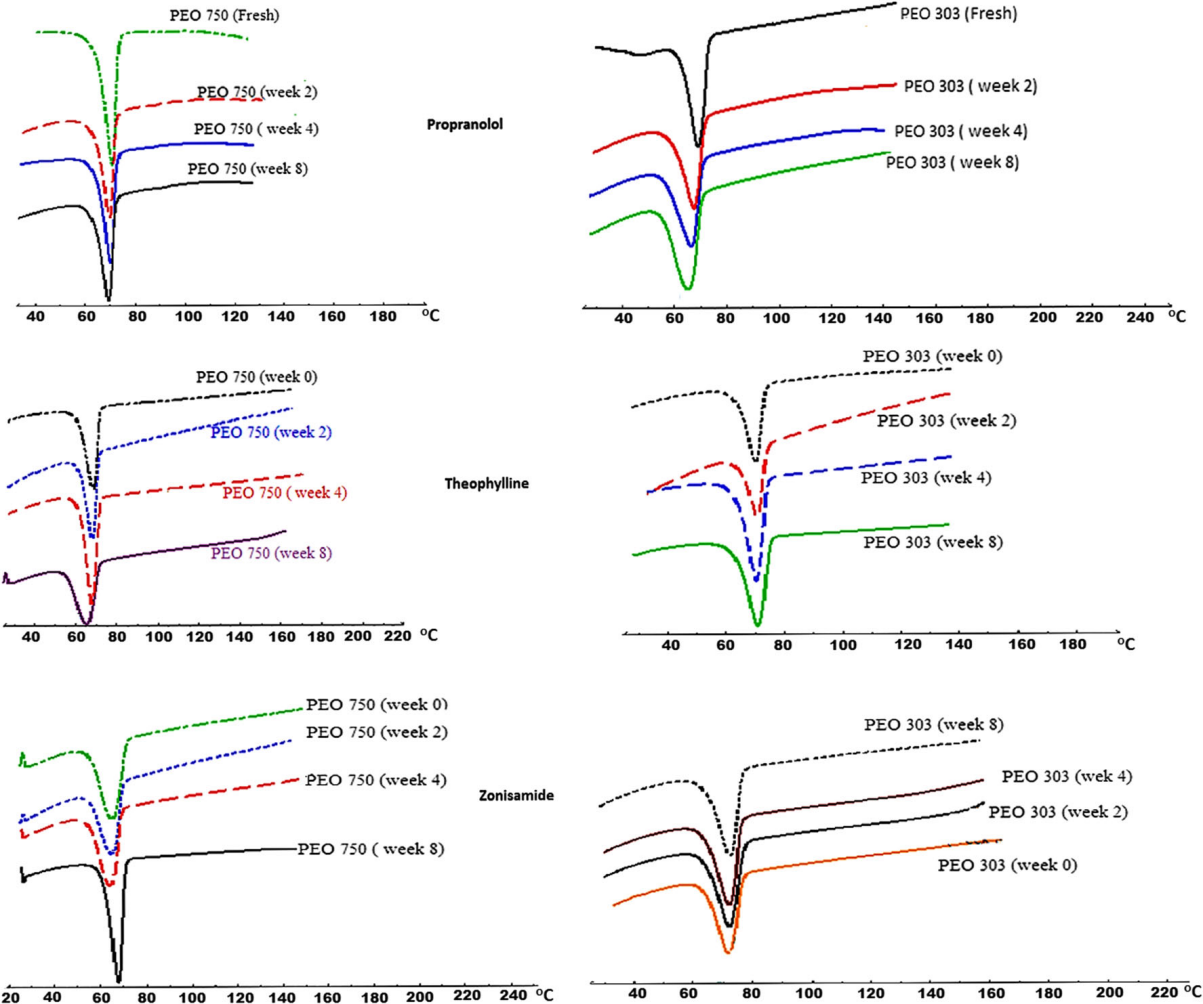
DSC thermograms for ground propranolol, theophylline and zonisamide matrix tablets: PEO 750 and 303 before and after storage times of 0, 2, 4 and 8 weeks

The thermal behaviour of the fresh and aged ground tablet matrices was studied by DSC and the results are presented in Fig. 8 (Table 5 and Supp. Tables 3 and 4). As can be seen from Fig. 8, there was a shift in melting points of polyox towards the lower temperatures compared to the fresh samples when propranolol was used as a model drug. DSC traces were further analysed in terms of their enthalpies and the results are shown in Table 5. DSC traces clearly showed a major change in thermal transitions following the storage of the matrix tablets at 40 °C where the enthalpy values decreased as the storage time increased. This indicates that there is some sort of plasticisation effect on the aged sample compared to the fresh samples. The reduction in the enthalpy could be due to a reduction in viscosity (see Table 4) and a decrease in molecular weight. The GPC techniques have already demonstrated that the aged samples may have changed to a lower molecular weight polyox upon storage (see Fig. 2). The data in Table 5 also indicated that there were major reductions in the temperature of the onset and peak of the melting endothermic for PEO grades investigated. The reduction in temperature or enthalpy became more pronounced as storage time was increased from 2 to 8 weeks.

**Table 5:** DSC parameters of various PEO ground propranolol matrix tablets at different storage times (0, 2, 4 and 8 weeks)

PEO grade	Time (week)	Enthalpy (J/g)	Onset (°C)	Peak (°C)
303	Fresh 2 weeks 4 weeks 8 weeks	−167.0 $\pm 1.0$	65.0$\pm 0.5$	71.0$\pm 1$.0
		−155.0$\pm 0.1$	63.0$\pm 1.0$	69.5$\pm 1$.0
		−142.0$\pm 1$.0	62.9$\pm 0.6$	69.0$\pm 0.5$
		−135.0$\pm 1$.0	61.5$\pm 0.4$	68.1$\pm 0.3$
750	Fresh	−133.0$\pm 0.1$	64.1$\pm 0.1$	70.0$\pm 0.2$
	2 weeks	−126.0$\pm 0.2$	63.5$\pm 1$.0	69.3$\pm 0.4$
	4 weeks	−119.0$\pm 0.3$	62.0$\pm 0.3$	69.0$\pm 1$.0
	8 weeks	−114.5$\pm 0.5$	60.0$\pm 0.7$	67.7$\pm 0.3$

For theophylline, it is interesting to note that in the case of aged polyox 303 no changes in the melting points of aged samples were observed compared to the fresh samples (Fig. 8 and Supp. Table 3). This could be the reason for similar dissolution profiles for fresh and aged polyox 303 matrices. This indicates that degradation did not occur for aged polyox 303 samples due to the presence of the less water-soluble drug, theophylline. The above finding was also confirmed by the enthalpy data reported in Supp. Table 3. It is clear from the table that in the case of polyox 750, the enthalpy values decreased as the storage time progressed but this was not the case for polyox 303 samples as similar enthalpies were obtained. This indicates that aged polyox 303 samples may retain their molecular weight in the presence of theophylline.

In contrast, zonisamide showed a different thermal event compared to that of other two model drugs. The results obtained from the DSC traces showed that there was no significant reduction in peak melting temperatures upon storage for both polyox 750 and 303. This suggests that there was no noteworthy shortening in polymer chain length through aerobic auto-oxidation [34]. DSC traces also clearly demonstrated that the extensive changes were not visible in any of the polyox grades used (Fig. 8). These results are confirmed by the DSC data in Supp. Table 4 which includes the enthalpy, onset and melting peak data of all zonisamide based samples. It is evident from the Supp. Table 4 that the solubility of zonisamide in the samples prevented the big reduction in the enthalpies of the polyox as it occurred for the samples containing highly soluble drug propranolol HCl.

## Conclusions

Various studies relating directly to the investigation of PEOs have suggested that a particular advantage of these polymers may be the rapid gelling of the polymer upon hydration followed by a significant swelling of the tablet system. However, when matrices made from fresh PEO are stored at elevated temperature, a significant degradation and depolymerisation as a result of storage time may occurred on polymer structure which was evident by dissolution profiles, GPC, viscosity data and DSC traces in the present study. The reduction in molecular weight progressed with time throughout the study and could be clearly observed using GPC and DSC analyses. Generally, the changes in molecular weight led to dramatic increases in the release rate of the water-soluble drug (propranolol HCl). After 8 weeks of storage, all the release profiles including those for the higher molecular weight grade PEO 303 clearly exhibited an increased release profile. Whereas these changes in the drug release were only observed in the case of polyox 750 when medium water-soluble drug (theophylline) was used. On the other hand, polymer formulations including semi soluble drug theophylline with PEO 303 and zonisamide as a poorly soluble drug containing either polyox 750 or 303 showed a stable drug release for 8 weeks. It can be concluded that the molecular weight and the solubility of drugs played major role in controlling the drug release from both the fresh and aged polyox matrices. As a futuristic approach, it will be worth examining the effect of a range of controlled relative humidity while the samples are stored at accelerated temperature over a prolonged period of time. Therefore, the explorative study will provide a detailed experimental gateway of the effect of temperature, storage time and humidity on the stability of PEO matrix tablets.

## Electronic supplementary material

ESM 1 (DOCX 11 kb)

ESM 2 (DOCX 11 kb)

ESM 3 (DOCX 12 kb)

ESM 4 (DOCX 12 kb)
