# Supplementary material for: Evaluation of the drug solubility and rush ageing on drug release performance of various model drugs from the modified release polyethylene oxide matrix tablets
Source: Drug Deliv Transl Res. 2016 Nov 21;7(1):111–24. doi: 10.1007/s13346-016-0344-5 (PMC5222914; doi:10.1007/s13346-016-0344-5)
Supplement: Supplementary file 1 — (DOCX 11 kb) [file 13346_2016_344_MOESM1_ESM.docx]

**Supp. Table 1.** Effect of storage time on dissolution parameters of theophylline PEO tablet matrices, (DE= Dissolution efficiency, MDT= Mean dissolution time).

| **PEO** | **Time (week)** | **DE (%)** | **MDT (h)** |
| --- | --- | --- | --- |
| **750** | **Fresh** | 80.0 ± 2.48 | 2.35± 0.03 |
| **750** | **2 weeks** | 85.0 ± 3.34 | 1.83± 0.39 |
| **750** | **4 weeks** | 86.0 ± 1.29 | 1.74±0.04 |
| **750** | **8 weeks** | 90.0 ± 0.16 | 1.36± 0.02 |
| **303** | **Fresh** | 34.0 ±4.03 | 6.08±1.30 |
| **303** | **2 weeks** | 33.48 ± 3.90 | 5.90± 0.54 |
| **303** | **4 weeks** | 36.0 ±3.15 | 5.01±0.66 |
| **303** | **8 weeks** | 33.0 ± 3.49 | 5.76± 0.43 |
