# Supplementary material for: Evaluation of the drug solubility and rush ageing on drug release performance of various model drugs from the modified release polyethylene oxide matrix tablets
Source: Drug Deliv Transl Res. 2016 Nov 21;7(1):111–24. doi: 10.1007/s13346-016-0344-5 (PMC5222914; doi:10.1007/s13346-016-0344-5)
Supplement: Supplementary file 2 — (DOCX 11 kb) [file 13346_2016_344_MOESM2_ESM.docx]

**Supp. Table 2.** Effect of storage time on dissolution parameters of zonisamide PEO tablet matrices, (DE= Dissolution efficiency, MDT= Mean dissolution time).

| **PEO** | **Time (week)** | **DE (%)** | **MDT (h)** |
| --- | --- | --- | --- |
| **750** | **Fresh** | 84.0 ± 3.45 | 1.96± 0.28 |
| **750** | **2 weeks** | 85.0 ± 1.38 | 1.92 ± 0.43 |
| **750** | **4 weeks** | 86.0 ± 2.00 | 1.66± 0.20 |
| **750** | **8 weeks** | 87.0 ± 2.24 | 1.60 ± 0.28 |
| **303** | **Fresh** | 35.0 ± 5.88 | 4.11± 0.16 |
| **303** | **2 weeks** | 38.0 ± 1.38 | 3.56± 0.43 |
| **303** | **4 weeks** | 39.0 ± 3.71 | 3.85± 0.56 |
| **303** | **8 weeks** | 28.0 ± 1.90 | 4.16 ± 0.27 |
