# Supplementary material for: Evaluation of the drug solubility and rush ageing on drug release performance of various model drugs from the modified release polyethylene oxide matrix tablets
Source: Drug Deliv Transl Res. 2016 Nov 21;7(1):111–24. doi: 10.1007/s13346-016-0344-5 (PMC5222914; doi:10.1007/s13346-016-0344-5)
Supplement: Supplementary file 3 — (DOCX 12 kb) [file 13346_2016_344_MOESM3_ESM.docx]

**Supp. Table 3.** DSC parameters of various PEO-theophylline ground theophylline matrix tablets at different storage times (0, 2, 4, and 8 weeks).

| **PEO Grade** | **Time (week)** | **Enthalpy (J/g)** | **Onset (°C)** | **Peak (°C)** |
| --- | --- | --- | --- | --- |
| **303** | **Fresh** | -165.0$\pm1$.0 | 64.0$\pm0.1$ | 71.0$\pm0.2$ |
|  | **2 - weeks** | -164.1$\pm2$.0 | 63.0$\pm1.0$ | 70.2$\pm1.0$ |
|  | **4 - weeks** | -163.5 $\pm1$.0 | 62.6$\pm0.5$ | 70.3$\pm0.5$ |
|  | **8 - weeks** | -163.0$\pm1$.0 | 61.0$\pm0.3$ | 70.5$\pm0.3$ |
| **750** | **Fresh** | -131.7$\pm2$.0 | 62.0$\pm1$.0 | 69.0$\pm0.1$ |
|  | **2 - weeks** | -126.5$\pm1$.0 | 61.5$\pm1$.0 | 68.$5\pm0.5$ |
|  | **4 - weeks** | -119.5$\pm1$.0 | 60.0$\pm0.5$ | 67.4$\pm0.5$ |
|  | **8 - weeks** | -113.2$\pm1$.0 | 59.0$\pm0.3$ | 67.0$\pm1$.0 |
