# Supplementary material for: Evaluation of the drug solubility and rush ageing on drug release performance of various model drugs from the modified release polyethylene oxide matrix tablets
Source: Drug Deliv Transl Res. 2016 Nov 21;7(1):111–24. doi: 10.1007/s13346-016-0344-5 (PMC5222914; doi:10.1007/s13346-016-0344-5)
Supplement: Supplementary file 4 — (DOCX 12 kb) [file 13346_2016_344_MOESM4_ESM.docx]

**Supp. Table 4.** DSC parameters of various PEO ground zonisamide matrix tablets at different storage times (0, 2, 4, and 8 weeks).

| **PEO Grade** | **Time (week)** | **Enthalpy (J/g)** | **Onset (°C)** | **Peak (°C)** |
| --- | --- | --- | --- | --- |
| **303** | **Fresh** | -170.0±1.0 | 64.3±1.0$(\pm1)$ | 71.0±1.0 |
|  | **2 - weeks** | -169.2±0.50$(\pm0.5)$ | 54.0±1.0$(\pm1)$ | 70.5$(\pm0.5)$±0.5 |
|  | **4 - weeks** | -168.5±0.3$(\pm0.25)$ | 56.0±2.0 | 70.1$(\pm0.6)$±0.6 |
|  | **8 - weeks** | -169.7±0.6$168.0(\pm0.6)$ | 57.3±1.0 | 70.8$.8(\pm1)$±1.0 |
| **750** | **Fresh** | -136.0±1.0 | 61.8$(\pm2)$±2.0 | 68.0±0.1 |
|  | **2 - weeks** | -134.0$(\pm0.3)$±0.3 | 60.0$(\pm0.5)$±0.5 | 67.6±1.0 |
|  | **4 - weeks** | -132.5$(\pm1)$±1.0 | 59.6±1.0 | 67.3$(\pm0.4)$±0.4 |
|  | **8 - weeks** | -131.7$\pm(0.2)$±0.2 | 58.0±0.5$(\pm0.5)$ | 66.8$.8(\pm0.3)$±0.3 |
